# Combination of Antiretroviral Drugs and Radioimmunotherapy Specifically Kills Infected Cells from HIV-Infected Individuals

**DOI:** 10.3389/fmed.2016.00041

**Published:** 2016-09-26

**Authors:** Dina Tsukrov, Alicia McFarren, Alfred Morgenstern, Frank Bruchertseifer, Eugene Dolce, Miroslaw K. Gorny, Susan Zolla-Pazner, Joan W. Berman, Ellie Schoenbaum, Barry S. Zingman, Arturo Casadevall, Ekaterina Dadachova

**Affiliations:** ^1^Albert Einstein College of Medicine, Bronx, NY, USA; ^2^European Commission, Joint Research Centre, Institute for Transuranium Elements, Karlsruhe, Germany; ^3^New York University School of Medicine, New York, NY, USA; ^4^Veterans Affairs New York Harbor Healthcare System, New York, NY, USA

**Keywords:** antiretroviral therapy, HIV, radioimmunotherapy, gp41, bismuth-213, patients

## Abstract

Eliminating virally infected cells is an essential component of any HIV eradication strategy. Radioimmunotherapy (RIT), a clinically established method for killing cells using radiolabeled antibodies, was recently applied to target HIV-1 gp41 antigen expressed on the surface of infected cells. Since gp41 expression by infected cells is likely downregulated in patients on antiretroviral therapy (ART), we evaluated the ability of RIT to kill ART-treated infected cells using both *in vitro* models and lymphocytes isolated from HIV-infected subjects. Human peripheral blood mononuclear cells (PBMCs) were infected with HIV and cultured in the presence of two clinically relevant ART combinations. Scatchard analysis of the 2556 human monoclonal antibody to HIV gp41 binding to the infected and ART-treated cells demonstrated sufficient residual expression of gp41 on the cell surface to warrant subsequent RIT. This is the first time the quantification of gp41 post-ART is being reported. Cells were then treated with Bismuth-213-labeled 2556 antibody. Cell survival was quantified by Trypan blue and residual viremia by p24 ELISA. Cell surface gp41 expression was assessed by Scatchard analysis. The experiments were repeated using PBMCs isolated from blood specimens obtained from 15 HIV-infected individuals: 10 on ART and 5 ART-naïve. We found that ^213^Bi-2556 killed ART-treated infected PBMCs and reduced viral production to undetectable levels. ART and RIT co-treatment was more effective at reducing viral load *in vitro* than either therapy alone, indicating that gp41 expression under ART was sufficient to allow ^213^Bi-2556 to deliver cytocidal doses of radiation to infected cells. This study provides proof of concept that ^213^Bi-2556 may represent an innovative and effective targeting method for killing HIV-infected cells treated with ART and supports continued development of ^213^Bi-2556 for co-administration with ART toward an HIV eradication strategy.

## Introduction

HIV/AIDS continues to be an enormous global health burden with over 34 million infected people worldwide (WHO data). HIV-infected individuals now live much longer due to the suppression of the viral replication by antiretroviral therapy (ART). However, ART cannot kill infected cells, and HIV remains an incurable disease (1). Intensification of ART regimens failed to produce eradication of residual HIV-infected cells both systemically and in the central nervous system (2, 3). Thus, any strategy for curing HIV must include a method to eliminate virally infected cells.

Radioimmunotherapy (RIT) is used in cancer treatment and is FDA-approved for treatment of non-Hodgkin’s lymphoma (4, 5). RIT uses monoclonal antibodies (mAbs) to target antigens over-expressed by tumor cells. The radiolabeled antibody acts as a homing device for the delivery of cytocidal ionizing radiation to cells expressing the targeted antigen. We have introduced RIT for a number of infectious diseases and demonstrated its feasibility for killing HIV-infected human cells *in vivo* (6, 7). Recently, we identified a fully human mAb 2556 directed toward a highly conserved epitope on the gp41 transmembrane glycoprotein, which is exposed both on viral particles and on the surface of infected cells. The 2556 mAb bound to the immunodominant domain (cluster 1) of gp41 shared across all subtypes within HIV clades A to H and was selected for preclinical development because of its superior binding to the gp41 when compared to naturally occurring antibodies in HIV-infected individuals. When radiolabeled with bismuth-213 (^213^Bi), an α-emitter with the 6–8 MeV energy of α-radiation, ^213^Bi-2556 killed HIV-infected human peripheral blood mononuclear cells (PBMCs) injected into SCID mice and produced no hematologic toxicity (8). Since the majority of HIV-infected individuals in the US are receiving ART and ART becomes more accessible to individuals worldwide, we have investigated the ability of ^213^Bi-2556 to kill HIV-infected cells from individuals on various ART regimens. Our results using *ex vivo* samples support further development of an RIT-based treatment for use with antiretroviral drugs toward HIV eradication.

## Materials and Methods

### Ethics Statement

All healthy blood donors provided written informed consent. All patients in the study were HIV-infected adults who provided written informed consent. The study was approved by the Montefiore Medical Center IRB #2011-1100.

### Compounds

Antiretroviral therapy drugs were selected to represent the three major ART classes typically prescribed for first-line therapy: nucleoside reverse transcriptase inhibitors (NRTIs), non-nucleoside reverse transcriptase inhibitors (NNRTIs), and protease inhibitors (PIs). The NIH AIDS Research and Reference Reagent Program provided all drugs, viral strains, and cell lines. The following four ART drugs were used: tenofovir (TFV, Cat. # 10199), emtricitabine (FTC, Cat. # 10071), efavirenz (EFV, Cat. # 4624), and atazanavir (ATZ) sulfate (Cat. # 10003). These compounds were dissolved in dimethyl sulfoxide (DMSO) to 10 ng/mL, serially diluted into phosphate-buffered saline (PBS), and stocks frozen at −20°C at 10× the desired final concentration. The drugs were assessed individually and in two clinically relevant combinations TFV/FTC/EFV and TFV/FTC/ATZ as per the guidelines for ART (9). The following cell lines and biological reagents from the same source were used: the A3.01 cell line, a human T-cell line derived from acute lymphoblastic leukemia (Cat. # 166, from Dr. Thomas Folks); the ACH-2 cell line (Cat. # 349 from Dr. Thomas Folks), an A3.01 subclone chronically infected with a single integrated copy of proviral LAV HIV_IIIB_; human recombinant IL-2 (Cat. # 136 from Dr. Maurice Gately); HIV-1 Ada-M (Cat. #416 from Dr. Howard Gendelman); and HIV-1 pNL4-3 (Cat. #114 from Dr. Malcolm Martin).

### Cellular Models of HIV Infection

ACH-2 and A3.01 cells were cultured in RPMI 1640 (Hyclone) with 2-mM glutamine, 10-mM HEPES, 1% (v/v) penicillin–streptomycin (Gibco-Invitrogen), and 10% (v/v) heat-inactivated fetal bovine serum (Hyclone). Phorbol 12-myristate 13-acetate (PMA, Sigma) was added to media containing 10^6^ cells/mL at 200 nM for 48 h to induce high viral production in ACH-2 cells (10). Activated ACH-2 cells were combined with the uninfected parental A3.01 line at 1:10 to simulate 10% infection in a sample. Human PBMCs were isolated using a Ficoll-Hypaque density gradient from healthy HIV-seronegative donors blood purchased from the New York Blood Center (New York, NY, USA) and grown in the media described above, replacing 10% FBS with 5% FBS and 10% human serum (Lonza). There are several reasons why we started with the cells from the healthy donors. First, we needed to study how the single ART drugs and then their combinations affect the gp41 expression on the cells. In real life, the patients are treated with at least triple combinations of drug, so that it would not be possible to delineate the effect of each drug. Second, we needed to investigate if the viral tropism affects the interaction between ART and gp41 expression. Isolated PBMCs at 2 × 10^6^ cells/mL were stimulated with phyto-hemagglutinin (PHA, Sigma) at 5 μg/mL and IL-2 at 10 U/mL for 48 h and then exposed for 3 h to either R5 HIV_ADA_ or X4 HIV_NL4-3_ virus at 25 ng/mL as in Ref. (11). Cells were resuspended in fresh media with IL-2 and grown for 48 h at 37°C in 5% CO_2_ for infection to spread. Cells exposed to HIV are referred to as “infected” cells and those not exposed to the virus as “non-infected” cells as per the methodology described in our previous studies (6, 8).

### Suppression of HIV Replication with ART

Peripheral blood mononuclear cells were cultured in a panel of ART drugs to titrate for optimal drug concentrations and show a dose–response of decreased HIV production with more drugs. TFV and FTC were initially tested at 0.001, 0.01, 0.1, 1, 10, and 100 μM with the goal of spanning the entire range of potential drug concentrations. The ranges were derived from the EC50 values for each of the drugs, 0.04–8.5 μM for TFV and 0.0013–0.64 μM for FTC (9). Subsequent experiments were performed using the middle concentrations of 1 and 10 μM, which caused moderate to complete inhibition of HIV replication *in vitro*. For combination treatments, molarities of TFV and FTC were scaled relative to the typically prescribed milligram drug ratios, using 6:3:2 molar ratios of TFV/FTC/ATZ and 3:3:2 molar ratios of TFV/FTC/EFV. Final DMSO concentrations were below 0.5% per sample, which has no effect on the yield of infectious virus or cell growth rate (12). Freshly thawed ART drugs were added to 1 mL of media containing 10 IU/mL IL-2 and 10^6^ cells. Cells were grown in low adhesion 24-well plates (Corning) in the presence of ART drugs for 6 days prior to treatment with ^213^Bi-2556, with the fresh media containing ART drugs and IL-2 given to cells on day 3.

### Radiolabeling of 2556 mAb

Clinical grade mAb 2556 against HIV-1 envelope glycoprotein gp41 was obtained from Goodwin Biotechnologies (Plantation, FL, USA). Control human mAb 1418 to parvovirus capsid VP1 described in Ref. (13) was produced in the laboratory of Drs. Susan Zolla-Pazner and Miroslaw K. Gorny. The 2556 and 1418 mAbs were conjugated to the chelating agent C-functionalized trans-cyclohexyldiethylenetriamine pentaacetic acid derivative (CHX-A″-DTPA) (Macrocyclics). ^213^Bi was obtained from a ^225^Ac/^213^Bi generator produced at the Institute for Transuranium Elements (Karlsruhe, Germany) as in Ref. (14). The 2556 and 1418 mAbs in carbonate buffer at pH 8.5 were incubated overnight at room temperature with CHX-A″-DTPA using 10-fold excess of CHX-A″-DTPA over mAb on a molar basis (e.g., 6.67 × 10^−8^ mol CHX-A″-DTPA over 6.67 × 10^−9^ mol mAb) and purified into 0.15M ammonium acetate buffer at pH 6.5 using Vivaspin concentrators (Sartorius). Radiolabeling of 2556 with ^213^Bi was accomplished by eluting ^213^Bi from the generator with 1 mL 0.1M HI solution, adjusting the pH of the eluate to 4.5 with 2.5M ammonium acetate, incubating ^213^Bi containing solution with the CHX-A″-DTPA-conjugated mAbs for 5 min at 37°C and quenching the reaction by adding 4 μL of 0.01M EDTA. The radiolabeling yields and purity were >95% as determined by instant thin layer chromatography (ITLC). The immunoreactivity of the radiolabeled 2556 was determined by gp41 ELISA as in Ref. (8) and was >90% of the immunoreactivity of the naïve 2556. The specific activity of the radiolabeled mAbs was kept constant at 185 MBq/mg antibody throughout the study.

### Quantification of gp41 Expression on ART-Treated Infected Cells

The Scatchard transformation of the binding data was performed as described in Ref. (8, 15) to quantify gp41 expression on infected PBMCs cultured in ART. We radiolabeled 2556 mAb with ^188^Re in place of ^213^Bi to avoid killing of cells by ^213^Bi during the binding and the difficulties in correctly measuring radioactivity several times during the experiment as ^213^Bi quickly decays with the half-life of 46 min. Five million cells per condition were incubated with increasing quantities of ^188^Re-2556 mAb (from 0.45 to 3 ng mAb per sample). The ratio of bound to unbound radioligand was plotted to identify the number of cellular binding sites from the *x*-intercept of the transformed Scatchard plot and the Ka constant from the ratio of *y* and *x* intercepts. The immunoreactivity of the radiolabeled antibody used in Scatchard experiments was >90% of the naïve 2556 as determined by gp41 ELISA (8). The experiments were performed four times with every sample in triplicate. FACS was used to confirm gp41 expression by staining 10^6^ cells with LIVE/DEAD Fixable Violet Dead Cell Stain Kit (Invitrogen) and with 2556 mAb at 20 μg/mL concentration directly labeled with Alexa Fluor 633 Protein Labeling Kit (Invitrogen).

### Treatment of ACH-2 Cells and PBMCs with ^213^Bi-2556 mAb

Following 6 days of culture in the presence of ART, the population from each sample well was resuspended in PBS and split equally between three 1.5-mL microcentrifuge vials (Fisher Scientific) with 330,000 cells/vial as in Ref. (6, 8). The first two vials in each set were treated with varying activities of ^213^Bi-2556 mAb (0–0.370 MBq/mL) and the third vial with an equivalent volume of PBS (“untreated”). After addition of the radiolabeled mAb, the total volume of each sample was adjusted to 1 mL with PBS. When applicable, the irrelevant mAb 1418 (13) labeled with ^213^Bi was used as a control to measure non-specific killing. The cells were irradiated in pellets which were formed by cells settling under the gravity. After 3 h at 37°C, the tubes with the cells were spun, unbound antibody removed (at this stage, almost all ^213^Bi has decayed), and the cells resuspended in their appropriate media with IL-2 (PBMCs) or PMA (ACH-2 cells) and cultured in 96-well plates (Fisher Scientific) at ~300,000 cells per well for 72 h at 37°C. Media did not contain ART drugs to allow for recovery of viral production by any surviving HIV-infected cells. All drug conditions were performed in duplicate and controls in triplicate.

### Assessment of *In Vitro* ^213^Bi-2556 Efficacy

The number of viable cells in the samples before and after treatment with ^213^Bi-2556 was assessed electronically using the Cellometer Auto 2000 Cell Viability Counter (Nexcelom Bioscience) and validated manually with a hemocytometer using the Trypan blue dye exclusion (16). Within each ART condition, the low-activity and high-activity ^213^Bi-2556 conditions were compared to 0 MBq to determine absolute and relative percent killing by ^213^Bi-2556, ART, and their combination. Viral titers in each well supernatant were measured by a well-validated (17) commercial double antibody sandwich enzyme immunoassay kit for HIV-1 p24 ELISA (Advanced Bioscience Laboratories), which detects the presence of the HIV-1 viral core p24 *gag* antigen with a detection limit of 3.1 pg/mL. The positive controls were serial dilutions of the manufacturer-provided HIV-1 p24 standard containing purified HIV-1 IIIB p24. Enzyme-linked absorbance was fit to a known HIV-1 IIIB p24 standard curve, and sample p24 concentration was determined from linear regression analysis. Samples were scored as undetectable when the measured absorption was below that of negative controls wells containing complete culture media.

### Statistical Analyses

Statistical significance for cell killing and p24 production was determined using a repeat measure two-way ANOVA comparing the significance of the RIT-induced effect within each ART drug condition relative to the “0 MBq Bi-2556” control wells for that condition, using the Dunnett adjustment for multiple comparisons. *P* = 0.05 was set *a priori* as the threshold for statistical significance. The multiplicity-adjusted *P* values were reported for each comparison.

### Selection of Patient Samples

Participants were recruited at the Montefiore AIDS Center outpatient clinic. HIV-infected PBMCs were isolated as described above from 40-mL blood samples following Montefiore Medical Center IRB #2011-1100. Five patients were recruited for each of the 2 combination ART regimens [TFV/FTC/EFV and TFV/FTC/ATZ/ritonavir (RTV)] along with 5 ART-naïve patients for a total of 15 patients. Within each ART group, three patients had well-controlled infections (undetectable HIV RNA) and two had poorly controlled infections (HIV RNA >1000 copies/mL). In the ART-naïve group, two patients had well-controlled infections (<10,000 copies/mL) and three had poorly controlled infections (>20,000 copies/mL). Patients with known substance abuse, resistance to their currently prescribed ART drugs, and elite controllers (<50 copies/mL sustained without ART) were excluded from the study.

### Assessment of *Ex Vivo* ^213^Bi-2556 Efficacy

Peripheral blood mononuclear cells were isolated from participants’ samples within 2 h of blood collection, treated with 0–0.74 MBq ^213^Bi-2556 mAb at 0–0.74 MBq/mL concentration, and analyzed in 3 days for cell survival using Trypan blue exclusion. Additionally, six participants’ samples were treated with radiolabeled control mAb 1418 (one patient from each ART-treated group and four from the ART-naïve group). Quantitative RT-PCR was used to determine viral levels immediately before ^213^Bi-2556 treatment and in the supernatants post-RIT. Samples were analyzed in duplicate on the Abbott m2000 RealTime HIV-1 assay PCR machine (Abbott Laboratories), which detects the integrase region of the HIV-1 *pol* gene down to 40 RNA copies/mL. The 100 μL of patient serum was used per sample. Machine readout was a numerical value, or undetectable RNA, or “RNA present but below detection limit,” which covers a range of 0–40 copies/mL. For the last category, values were conservatively estimated at the limit of detection (LOD) of 40 copies/mL which converted to 400 copies/mL. Values reported are the mean of two duplicate conditions, processed separately.

### Calculations of the Projected Human Dose of ^213^Bi-2556 mAb

We performed the calculations of the projected human dose of ^213^Bi-2556 mAb by two different methods. The first method utilized the results of treating *ex vivo* PBMCs from patients on various ART regimens. For the *ex vivo* calculations, we used the following numbers: every patient donated 40 mL of blood from which 5 × 10^7^ PBMCs were isolated. We used 3 × 10^5^ PBMCs per sample to treat with ^213^Bi-2556. The lowest activity of ^213^Bi-2556 to kill significant percentage of infected cells was 0.037 MBq; thus, to achieve this result for 5 × 10^7^ PBMCs, one needs (0.037 MBq × 5 × 10^7^/3 × 10^5^) = 6.17 MBq. An adult’s blood volume is approximately 5000 mL (5 L); so to treat the PBMCs in 5 L of blood, one will need (6.17 MBq × 5000/40) = 771 MBq ^213^Bi-2556. This number can be also expressed as MBq/kg, in which case for a 60-kg person, it will be 771/60 = 12.8 MBq/kg.

For comparison, we also performed the calculations using alternative strategy by using our previously published data on treating HIV in mouse models with ^213^Bi-2556 mAb (8). Interspecies scaling factor between mice and humans is equal to 12.3, which reflects 12.3-fold difference in surface area to body weight ratio for a mouse (0.0066 m^2^/0.02 kg) as compared to that for a human (1.6 m^2^/60 kg) (18). Consequently, 12.3 times more drug is required in the mouse to be comparable to the dose in humans. From our RIT of HIV mouse work, we know that HIV-infected hPBMCs in mice were eliminated in peritoneal model with 3.7 MBq ^213^Bi-2556 (mAb) (8). Thus, the radioactivity for a mouse per kg body weight is calculated as 3.7 MBq/0.02 kg = 185 MBq/kg. Accordingly, for a human taking into consideration 12.3 as conversion factor, it will be 185/12.3 MBq/kg = 15.0 MBq/kg. For a 60-kg human, the total radioactivity of ^213^Bi-2556 will be 60 × 15.0 = 900 MBq. To calculate the amount of “cold” 2556 mAb in the radiolabeled preparation, the calculations were performed in the same fashion as given above using the following numbers: mAb dose in a mouse 0.02 mg/0.02 kg = 1 mg/kg; mAb dose in a human 1/12.3 = 0.08 mg/kg; and total “cold” 2556 mAb dose for a 60-kg human 0.08 × 60 = 4.8 mg. All calculations assumed a specific activity of 185 MBq/mg antibody for the radiolabeled antibody, which was used in all experiments in this manuscript and in the previous work (8).

## Results

### Gp41 Is Present on ART-Treated HIV-Infected Cells

**Figure 1:**
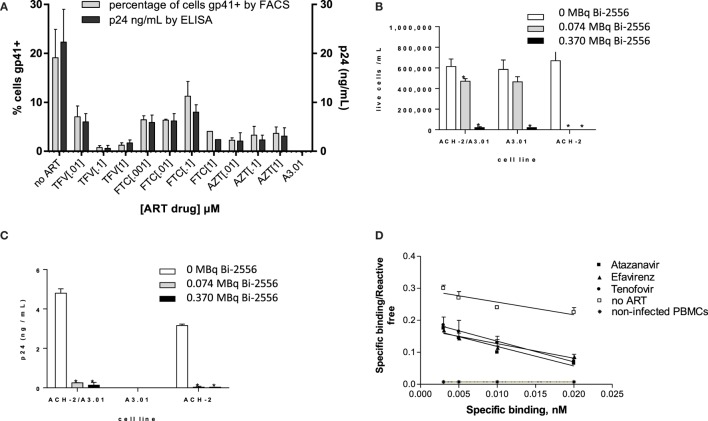
**2556 mAb binding and killing of HIV-infected cells lines**. **(A)** FACS analysis of 2556 binding to ACH-2 cells. Uninfected parental cell line A3.01 was used as a negative control. FTC – emtricitabine, TFV – tenofovir, and AZT – zidovudine; **(B)** ^213^Bi-2556 mAb treatment of stimulated ACH-2 cells and parental A3.01 cells, individually and in 1:10 combinations; and **(C)** p24 levels decreased post-RIT in both the combination and ACH-2 alone conditions, with no p24 detected in the control A3.01 samples. All cells were incubated with ^213^Bi-labeled mAbs for 3 h before being plated for survival. The error bars show SD; experiment was performed three times; * denotes statistically significant *P* value of <0.05 compared to 0 MBq in each ART group. The cell survival was measured 72 h post-treatment. **(D)** Scatchard analysis of ^188^Re-2556 binding to HIV_ADA_-infected PBMCs with and without ART treatment. No significant binding was detected on the uninfected PBMCs.

Our initial goal was to ascertain whether ART would decrease the gp41 expression on the surface of ART-treated infected cells. To the best of our knowledge, the expression of gp41 on ART-treated HIV-infected cells has not been quantified; however, given that ART interferes with viral replication, there is a concern that gp41 might be reduced (19) or even absent on the cell surface. We treated the ACH-2 human lymphocyte cell line (chronically infected with X4 HIV_IIIB_) with three major NRTIs and examined the gp41 expression by FACS and the viral load by p24 ELISA. The following drugs were used in biologically relevant concentrations (0.001–1.0 μM): TFV (adenosine analog), FTC (cytidine analog), the 3:2 combination of TFV:FTC (a common backbone of ART combination treatment), and zidovudine (AZT, thymidine analog). FACS analysis of ACH-2 cells after exposure to ART demonstrated that the number of gp41-positive cells in the ART-treated samples was 5–40% of the number of gp41-positive cells in untreated population, and this decrease correlated with decrease in viral p24 production (Figure 1A). This observation established that significant numbers of ART-treated cells remain gp41-positive. The ART drug treatments had no effect on autofluorescence compared to control cells, and therefore, the values are compared to uninfected control cells. This was supported by results of killing experiments in which the ACH-2 and parental HIV-negative A3.01 cells were treated individually, and in a 1:10 combination with 0, 0.074, and 0.37 MBq/mL ^213^Bi-2556 mAb. The ACH-2 cells were mixed with the parental non-infected cells A3.01 in 1:10 ratio to demonstrate the specificity of killing of infected ACH-2 by radiolabeled 2556 mAb versus non-infected cells. ^213^Bi-2556 killed significantly more infected ACH-2 cells than uninfected A3.01 cells (Figure 1B) and decreased p24 to undetectable levels (Figure 1C). It is important to point out that as 2556 mAb binds also to gp41 on the viral particles (8), the non-infected cells with some viral particles getting attached to their surface can also be killed by radiation, which results in increased overall cell kill. In addition, since this is the first study of the ART and RIT combination for treatment of the HIV-infected cells, we used a broad range of ART and RIT concentrations to identify the maximum tolerated doses. In case of RIT, 0.37 MBq/mL of ^213^Bi-2556 mAb was five times higher than 0.074 MBq/mL and produced some off-target toxicity.

### Quantification of gp41 Expression on the ART-Treated HIV-Infected Cells

Scatchard analysis of radiolabeled 2556 mAb binding was used to quantify gp41 expression on the surface of PBMCs infected with R5-tropic strain HIV_ADA_ and treated with TFV, FTC, the PI ATZ, and the NNRTI EFV. Scatchard analysis revealed that ART-treated cells display over 10^4^ gp41 binding sites per cell (Figure 1D): for ATZ-treated cells, the number was 5.7 ± 0.2 × 10^4^ per cell; for EFV, 7.0 ± 0.4 × 10^4^; for TFV, 4.0 ± 0.3 × 10^4^; and for FTC, 3.9 ± 0.4 × 10^4^. These numbers are in the 40–70% range of 1.0 ± 0.2 × 10^5^ binding sites on untreated cells. In cancer RIT studies where similar antigen levels on the surface of the targeted cells were observed *in vitro* (20, 21), the RIT was nevertheless successful in killing the targeted cells. Several sequential RIT administrations might be required, however, to eradicate all infected cells in an HIV patient. There was no significant binding of 2556 to uninfected PBMC controls. The binding constant for the 2556 binding to gp41 was not affected by the ART drugs and calculated to be 9.8 × 10^7^/M, which is very close to 1.1 × 10^8^/M as in Ref. (8). We concluded that gp41 expression on the surface of ART-treated cells is reduced but sufficient for the radiolabeled mAb to bind and deliver cytocidal radiation to the infected cells.

### No Significant Killing of Uninfected PBMCs by ^213^Bi-2556 or Infected PBMCs by Control mAb ^213^Bi-1418 Was Observed

**Figure 2:**
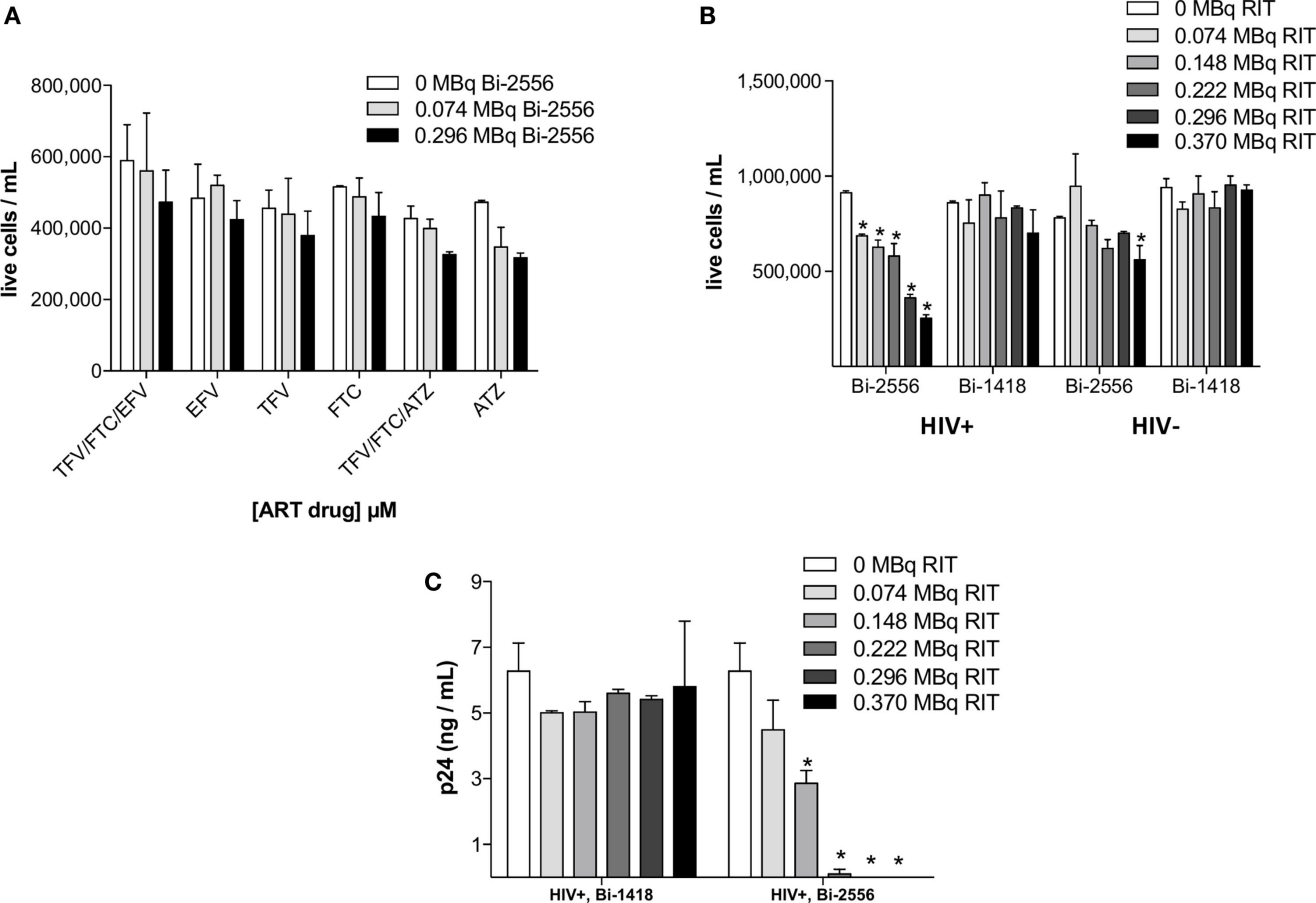
**PBMC-negative control experiments**. **(A)** Uninfected PBMCs were neither killed by ART alone nor by co-administration of ART and ^213^Bi-2556 at the maximum concentration of each drug (10 μM); **(B)** ^213^Bi-2556 treatment of infected PBMCs results in significantly higher killing than either ^213^Bi-2556 treatment of uninfected PBMCs or infected PBMCs treated with radiolabeled irrelevant 1418 mAb; and **(C)** ^213^Bi-2556 eliminates detectable p24 at higher concentrations, in contrast to ^213^Bi-1418. All cells were incubated with ^213^Bi-labeled mAbs for 3 h before being plated for survival. The cell survival was measured 72 h post-treatment. The error bars show SD; experiment was performed two times; * denotes statistically significant *P* value of <0.05 compared to 0 MBq in each ART group.

There was no statistically significant killing by ^213^Bi-2556 of uninfected ART-treated PBMCs (Figure 2A). This indicated that killing with ^213^Bi-2556 was HIV-specific and ART has neither radioprotective nor radiosensitizing effect. The specificity of ^213^Bi-2556 was further confirmed by the absence of killing of infected and uninfected PBMCs by ^213^Bi-labeled irrelevant 1418 mAb to parvovirus VP1 capsid protein (13) (Figures 2B,C). The 1418 is a fully human 1gG1 mAb and was used as a control mAb in our previous work on RIT of HIV-infected cells and showed no killing of HIV-infected cells (6, 8). ^213^Bi-2556 might possess some cross-reactivity with the cellular membrane proteins, which would result in it being “stickier” that ^213^Bi-1418 mAb and causing more killing of uninfected PBMCs than ^213^Bi-1418, though the difference was not statistically significant.

### ^213^Bi-2556 Killed ART-Treated PBMCs Infected with Different Viral Strains of HIV in a Dose-Dependent Manner

**Figure 3:**
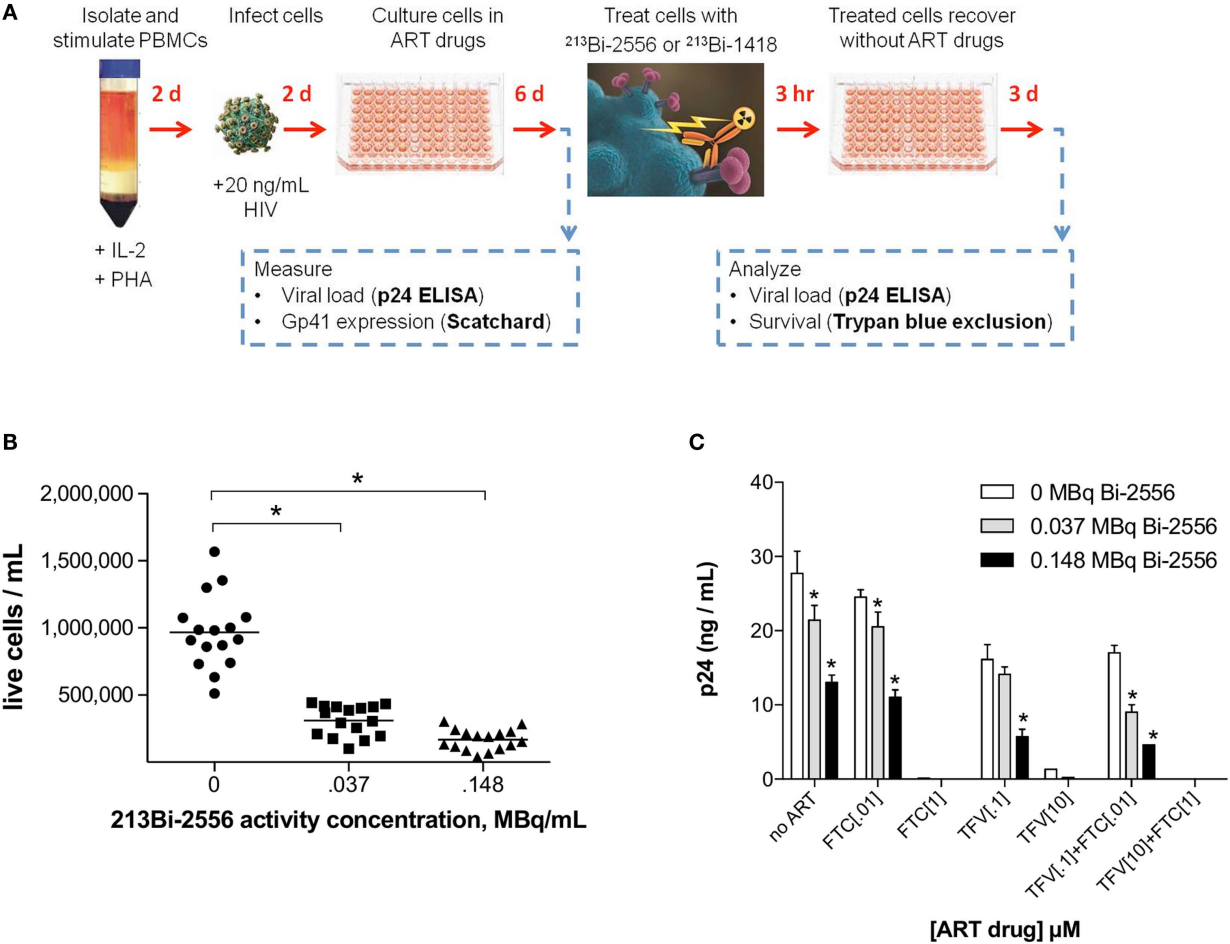
**Killing of PBMCs infected with X4 HIV_NL4-3_ strain and treated with NRTIs**. **(A)** Design of ART and RIT combination experiments; **(B)** post-RIT survival of PBMCs treated with TFV, FTC, or TFV:FTC = 3:2. Shown are averages of two duplicate conditions per ART concentration, five concentrations per ART drug. Sixteen replicates per RIT dose; **(C)** post-RIT p24 analysis of high and moderate ART conditions from each treatment group. All cells were incubated with ^213^Bi-labeled mAbs for 3 h before being plated for survival. The cell survival was measured 72 h post-treatment. The error bars show SD; experiment was performed three times; * denotes statistically significant *P* value of <0.05 compared to 0 MBq for each ART group.

**Figure 4:**
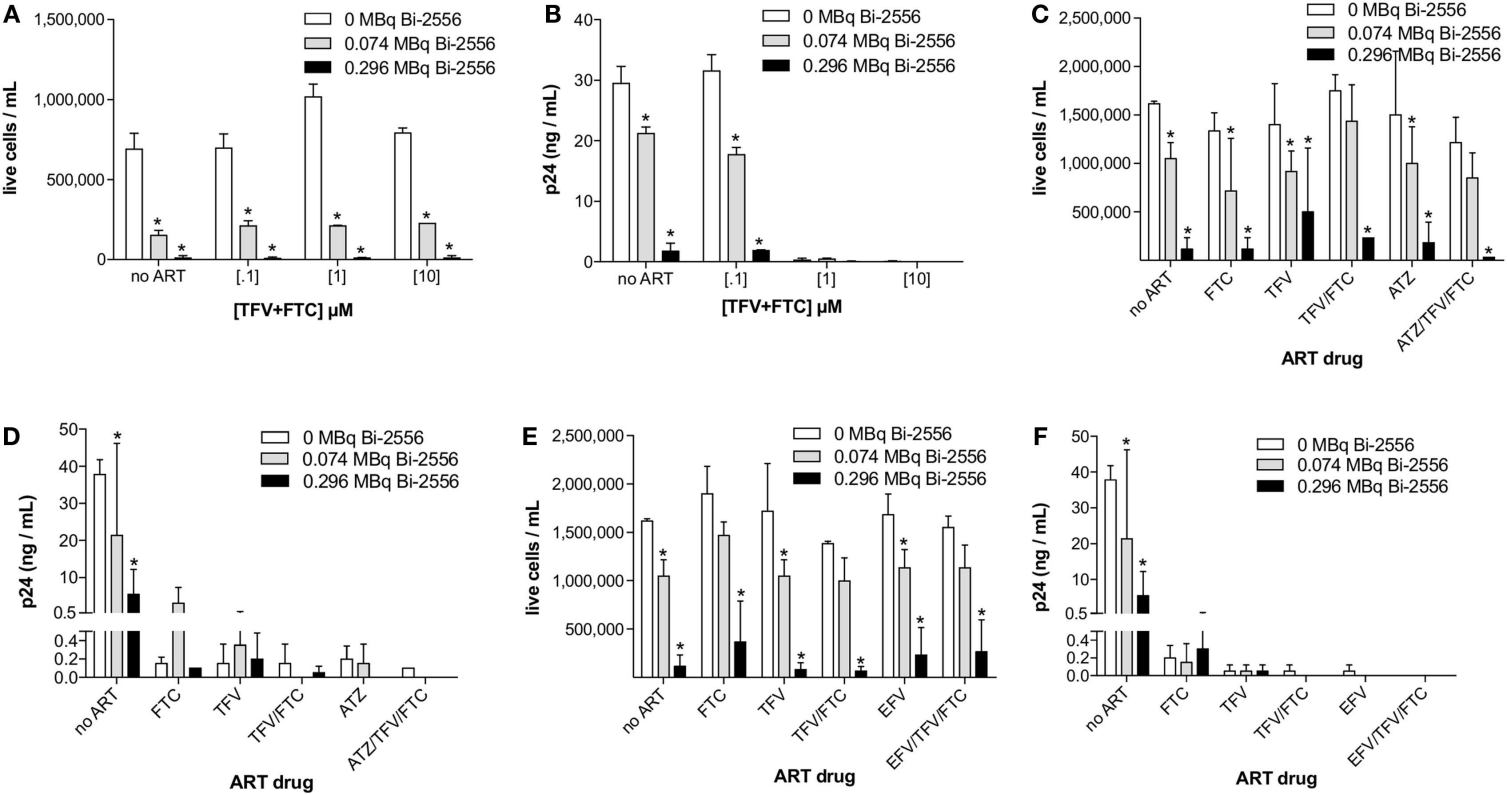
**Post-RIT killing and p24 analysis of R5 HIV_ADA_-infected PBMCs treated with 3:2 combinations of TFV:FTC (A,B) or with the addition of atazanavir (C,D) or efavirenz (E,F)**. Atazanavir and efavirenz were each used at 10 μM and the ATZ:TFV:FTC and EFV:TFV:FTC molarities in **(C–F)** were scaled relative to the typically prescribed ratios of 6:3:2 and 3:3:2, respectively. All cells were incubated with ^213^Bi-labeled mAbs for 3 h before being plated for survival. The cell survival was measured 72 h post-treatment. The error bars show SD; experiment was performed four times; * denotes statistically significant *P* value of <0.05 compared to 0 MBq in each ART group.

The experimental schema for these experiments is shown in Figure 3A. There are several reasons why we started with the cells from the healthy donors. First, we needed to study how the single ART drugs and then their combinations affect the gp41 expression on the cells. In real life, the patients are treated with at least triple combinations of drug, so that it would not be possible to delineate the effect of each drug. Second, we needed to investigate if the viral tropism affects the interaction between ART and gp41 expression and when the patients are recruited into the study, their viral strain is not known. On the contrary, we were able to infect the cells with either R5 or X4 viruses with different tropism. ^213^Bi-2556 killing of X4 HIV_NL4-3_-infected cells treated with FTC, TFV, or their combination occurred in a dose-dependent manner (Figures S1A–C in Supplementary Material), with the majority of infected cells in all ART conditions eliminated by 0.150 MBq/mL ^213^Bi-2556 mAb (Figure 3B). A corresponding decrease in viral load post-RIT was observed by p24 ELISA for all samples (Figure 3C). We also examined the RIT of cells infected with R5-tropic HIV_ADA_ strain. Dose-dependent cell killing by ^213^Bi-2556 was accompanied by considerable reduction in viral load for HIV_ADA_-cells treated with the FTC and TFV (Figures 4A,B). In cells exposed to higher doses of ^213^Bi-2556 and combined FTC/TFV, the post-treatment viral load was undetectable. It should be noted here that the absence of p24 in the supernatant is a clear indication that there are no infected cells left, as p24 is a fragile protein which cannot survive several days in culture medium at 37°C (22). To further simulate the clinically relevant ART drug combinations, we combined FTC and TFV with either ATZ (Figures 4C,D) or EFV (Figures 4E,F). HIV-infected cells in both combinations were killed in a dose-dependent manner; however, the combination with EFV was more effective than with ATZ in completely eliminating infected cells at higher doses of ART and ^213^Bi-2556. Taken together, these results demonstrated that for cells infected with different viral strains, pretreatment with ART does not impede the killing with ^213^Bi-2556 and provided the impetus for *ex vivo* RIT of PBMCs from HIV-infected individuals.

### ^213^Bi-2556 Treatment of *Ex Vivo* Patient Samples Was Effective in Killing Infected Cells and Decreasing the Viral Load

**Figure 5:**
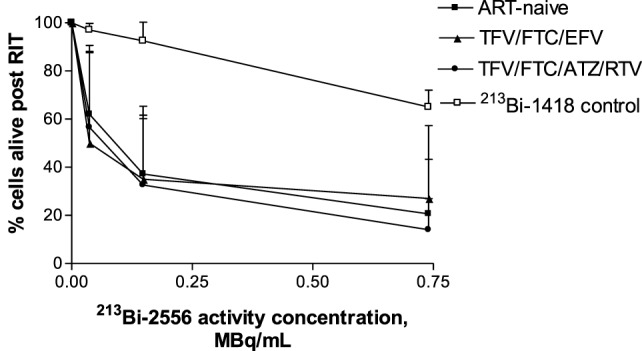
**Killing of PBMCs derived from ART-treated patients per treatment group (*n* = 5 per group) by ^213^Bi-2556 compared with killing by the ^213^Bi-labeled irrelevant control mAb 1418 (six replicates per condition were used)**. All cells were incubated with ^213^Bi-labeled mAbs for 3 h before being plated for survival. The cell survival was measured 72 h post-treatment. EFV combo – TFV/FTC/EFV; ATZ combo – TFV/FTC/ATZ/RTV.

Fifteen study participants were recruited including those with well- and poorly controlled infection: five for each of the two ART regimens TFV/FTC/EFV and TFV/FTC/ATZ/ritonavir (RTV) and five ART-naïve patients (Table 1). The three groups were similar in age: the mean age was 43.6, 42.4, and 40.6 years for the TFV/FTC/EFV, TFV/FTC/ATZ/RTV, and ART-naïve groups, respectively (*P* = 0.1). The mean time from the diagnosis was the 6.0, 9.8 and 11.0 years in ART-naïve, TFV/FTC/EFV and TFV/FTC/ATZ/RTV groups, respectively. The difference in the mean time from the diagnosis was significant between ART-naïve and TFV/FTC/EFV groups (*P* = 0.04) and between ART-naïve and TFV/FTC/ATZ/RTV groups (*P* = 0.02) but was not significant between the two ART-treated groups (*P* = 0.08). PBMCs isolated from the participants’ samples were treated with 0–0.74 MBq/mL ^213^Bi-2556 mAb and analyzed for cell survival and residual viral production (Figure S2A in Supplementary Material). Additionally, six samples were treated with 0–0.74 MBq/mL radiolabeled control mAb 1418 to ascertain the specificity of killing by ^213^Bi-2556 mAb. Dose-dependent killing of infected cells with ^213^Bi-2556 was observed for all participants in all three groups (Figures S2B–D in Supplementary Material). Overall, the treatment success was observed to be greater in the ART groups than in the ART-naïve group. However, we did not perform statistical comparisons between the groups due to the small number of study participants. The ^213^Bi-2556 mAb was specific as substantially less killing was seen with ^213^Bi-1418 control across all cohorts (Figure 5). Some killing seen with ^213^Bi-1418 was caused by random hits of emitted radioactive particles. The lymphocytes are some of the most radiosensitive cells in the body, and non-specific killing is unavoidable for *in vitro* assays performed in small volumes, but *in vivo* the side effects of ^213^Bi-2556 treatment were not observed (8). While side effects of RIT for cancer treatment may include transient and long-term myelodysplasia, neutropenia, and thrombocytopenia, the net safety record of RIT is strong particularly in comparison with the high morbidity and low success rates of HIV gene therapy approaches (23). An HIV-infected patient would be more vulnerable to the negative effects of myelosuppression than a healthy individual and should be monitored closely post-treatment, but the similarly vulnerable populations of cancer patients were treated with RIT with only transient myelosuppression (24, 25).

**Table 1:** **Demographic characteristics and HIV infection parameters of the study participants**.

Patient	Age	Gender	Years since diagnosis	CD4/mL	Viral RNA copies/mL
**ART-naïve**					
Well-controlled					
DT11	49	F	20	1418	951
DT15	27	F	1	416	2254
Poorly controlled					
DT04	27	M	4	986	57,301
DT08	51	M	1	304	30,876
DT09	49	M	4	682	48,558
**TFV/FTC/EFV**					
Well-controlled					
DT02	54	F	10	784	Not detected
DT07	34	M	1	549	Not detected
DT14	24	F	7	870	Not detected
Poorly controlled					
DT12	59	M	22	356	1287
DT13	47	M	9	468	2507
**TFV/FTC/ATZ/RTV**					
Well-controlled					
DT01	53	M	3	986	Not detected
DT05	45	F	15	669	Not detected
DT06	36	M	17	681	Not detected
Poorly controlled					
DT03	53	M	14	22	36,453
DT10	25	M	6	334	39,902

The data in Table 1 on patients’ treatment and viral loads served as the basis for patients’ selection for a particular cohort in the study. As the number of patients in the study was small, it would be impossible to make any correlations between the length the patients been on ART and their response to RIT. The patients with the undetectable viral loads are considered fully suppressed and there were six patients like this during the initial selection. Because the patients might experience lessening or improvement in viral control during the time past between their last blood test and RIT, we performed RT-PCR on their PBMCs immediately after their blood was donated for RIT and after the intermediate (0.15 MBq/mL) and high (0.74 MBq/mL) RIT activities (Table 2). The discrepancy between the well or poorly control status was observed for only for two patients – DT03 and DT15. Patient DT03 who was recruited as poorly controlled in TFV/FTC/ATZ/RTV cohort became well controlled probably due to the improved compliance, while a well-controlled ART-naïve patient DT15 experienced the lessening of the viral control, which is not unusual in ART-naïve patients. Importantly, the amount of virus detected in the treated samples decreased, often to undetectable levels, in 14 of 15 *ex vivo* samples (Table 2). No decrease in viral load was observed in the DT15 participant who was initially recruited as an ART-naïve with well-controlled viremia (Table 1). A possible explanation for the increase in viral RNA copies/mL post-RIT in this patient could be that the cells killed by ^213^Bi-2556 released viral particles as the cells were dying from radiation-induced apoptosis without ART drugs. This observation highlights the importance of continuing maintenance on ART during RIT to prevent the re-infection of cells by the released viral particles.

**Table 2:** **Patient viral levels post-RIT with ^213^Bi-2556 mAb (RNA copies/mL)**.^a^

Patient	0 MBq/mL	0.15 MBq/mL	0.74 MBq/mL
**ART-naïve**			
Well-controlled			
DT11	Not detected	Not detected	Not detected
DT15	31,860	50,770	35,355
Poorly controlled			
DT04	44,702	18,300	13,883
DT08	257,040	108,735	5480
DT09	113,670	138,075	4425
**TFV/FTC/EFV**			
Well-controlled			
DT02	288	Not detected	Not detected
DT07	<400^b^	Not detected	Not detected
DT14	Not detected	250	Not detected
Poorly controlled			
DT12	18,420	<400^b^	Not detected
DT13	8245	2795	325
**TFV/FTC/ATZ/RTV**			
Well-controlled			
DT01	Not detected	Not detected	<400^b^
DT05	310	Not detected	Not detected
DT06	Not detected	Not detected	Not detected
Poorly controlled			
DT03	<400^b^	<400^b^	<400^b^
DT10	49,775	14,890	Not detected

*Values reported are the mean of two duplicate conditions, processed separately*.

*^a^0, 0.15, and 0.74 MBq/mL ^213^Bi-2556 were used. The radiochemical purity of ^213^Bi-2556 mAb was 95%, the specific activity 185 MBq/mg, and the immunoreactivity 93%*.

*^b^For the marked samples, the Abbott m2000 readout “RNA present but below detection limit” was conservatively estimated at the limit of detection of 40 copies/mL and adjusted to 400 copies/mL with dilution factor*.

Finally, we calculated the activity of ^213^Bi-2556 mAb for an HIV-infected individual to achieve the same degree of infected cell killing as observed *ex vivo*. Assuming that an adult’s blood volume is 5 L, the projected activity will be 740 MBq, which for a 60-kg person will be 740/60 = 12.3 MBq/kg. We also performed an alternative calculation using our data on treating HIV in mouse models with ^213^Bi-2556 (8) and the interspecies scaling factor between mice and humans. The calculated activity of 910 MBq (15.2 MBq/kg for a 60-kg person) was close to the activity calculated from the *in vivo* results. Using the same approach, the amount of the mAb in preparation was calculated to be 0.08 mg/kg. It is important to say that most likely *in vivo* there will be no “cross-fire” killing of the separately localized infected cells, and this is why the activity administered to a patient needed for complete elimination of the infected cells might be significantly higher than calculated here or even will have to be given several times. Of note, the projected patient dose calculated from the mouse experiments is higher than that from the *in vitro* experiments. The question of specific activity is also very important when the projected patient dose is discussed. For a specific activity of 185 MBq/mg, only 1 antibody out of 5000 is radiolabeled, which means that 14 radiolabeled antibodies will be attached to each cell. This will result in seven particles crossing the cell but, on average, only one alpha particle will cross the nucleus (considering the probability of 0.067 to cross the nucleus when the cell is 10 μm in radius and the nucleus is 5 μm in radius). Thus, increasing specific activity of the radioconjugate might be crucial for the success of RIT in HIV patients. In this regard, our on-going experiments show that 2556 mAb can be very reproducibly radiolabeled with 740 MBq/mg specific activity without losing its immunoreactivity (Dina Tsukrov, unpublished observations). Finally, it should be noted that such calculations can serve only as a theoretical estimate to compare a projected dose for an HIV patient to that of a cancer patients. In real clinical situation, the individualized dose calculation will involve first imaging of a patient with immunoSPECT or immunoPET and taking into consideration his/her bone marrow reserve.

## Discussion

Here, we describe the first experience of using RIT to treat *ex vivo* PBMCs from ART-treated and ART-naïve individuals, to evaluate the killing efficacy of ^213^Bi-2556 with concurrent ART treatment. There was a concern that ART would reduce the expression of the gp41 target protein on the infected cells to such levels that the radiolabeled mAb would not bind; however, the Scatchard analysis of the 2556 binding to the infected and ART-treated cells demonstrated sufficient residual expression of gp41 on the cell surface to warrant subsequent RIT. To the best of our knowledge, this is the first time the quantification of gp41 post-ART is being reported. Our results are indirectly confirmed by the recent report by Santangelo et al. who detected gp120 expression on the SIV-infected cells *in vitro* and *in vivo* in averimic SIV-infected macaques on ART and in elite controllers alike (26).

^213^Bi-2556 alone eliminated most, but not all, of the detectable viral production according to p24 ELISA; however, the combination of ART and RIT completely eliminated detectable virus. Our results suggest that RIT would be most effective in the presence of ART. An additional benefit of using ART is the reduction of free virus in the blood stream (which would otherwise present additional gp41 targets) freeing the ^213^Bi-2556 to seek and kill infected cells. The killing results indicate that even when gp41 expression is reduced by ART, there are binding sites on the surface of infected cells for the radiolabeled mAb to bind and kill HIV-infected cells. This observation is supported by cancer RIT data for cells with similar levels of antigen expression (20, 21, 25) and by RIT of *Bacillus anthracis* bacterial infection (27). A few alpha particles transversing a nucleus are considered capable of killing a cell (28, 29). It should be noted that percentage of HIV-infected PBMCs in patients ranges from 0.6 to 20% (30). Due to the stationary nature of the killing assays utilized in this study, up to 30% of non-specific killing of PBMCs was observed in patients’ samples at the highest activity of 0.75 MBq/mL as measured with control mAb ^213^Bi-1418 (Figure 5). Such non-specific killing was not observed *in vivo* in mice carrying HIV-infected human cells and treated with RIT (6, 8).

Elimination of HIV-infected cells will necessarily constitute a backbone of any HIV eradication strategy. Currently, the dominant experimental strategy to eliminate infected cells is reactivation of latent HIV using agents such as histone deacetylase (HDAC) inhibitors (31). However, it is still unclear if such reactivation, accompanied by aggressive ART or other treatments, will result in eradication of all the infected cells or in new infections. Gene disruption and bone marrow transplantation are investigated for their potential to cure HIV (32, 33), but these are risky and expensive procedures that are currently not feasible for the treatment of large numbers of patients. The success in oncology of naked mAb therapy, immunotoxins [e.g., FDA-approved trastuzumab drug conjugate for metastatic breast cancer (34)], and RIT contributed to the renewed interest in HIV elimination strategies based on armed mAbs (35–37). The advantages of armed antibodies approach for HIV cure is the independence from the host immune system. Also, antibody–antigen interaction is not subject to multidrug resistance mechanisms. Significant depletion of the systemic viral reservoir in BLT mice from combined ART and immunotoxin treatment was reported (38), although the challenges of complex chemistry and inherent immunogenicity remain to be resolved. A fully human mAb ^213^Bi-2556 used in this study possesses additional advantages of not being immunogenic, not requiring internalization, and having minimal toxicity due to ^213^Bi 46 min half-life resulting in decay of a projected 740 MBq human dose to nearly undetectable levels (1% or 7.4 MBq) within 5 h of administration. ^213^Bi-labeled mAbs and peptides are in clinical trials for several oncological indications, both as the primary therapy and in combination with prior chemotherapy (39–41). The projected activity for a human of 12.3 MBq/kg is close to ~1.0 GBq cumulative activity of ^213^Bi-HuM195 mAb given to patients with acute myeloid leukemia (39). Generally, injection of ^213^Bi immunoconjugate induces some hematologic toxicity in patients, for example, on leukocyte and red blood cell (42, 43). It remains to be seen if the lack of toxicity *in vitro* in this study will be observed in patients as well.

The use of targeted therapy with α-particle emitters is burgeoning worldwide, driven by the advantages of α-emitters over β-emitters including specific targeting of the diseased cells due to the α-particles’ short 50–80 μm tissue range and increased killing efficiency due to high linear energy transfer (44). It has been demonstrated experimentally that RIT with α-emitters does not depend on the oxygenation status of the tumor or its resistance to chemo and external beam radiation therapy (45, 46) and later confirmed clinically by achieving ^213^Bi-labeled peptide-induced remission in patients with neuroendocrine tumors refractory to β-radiation (43). Clinical trials of ^213^Bi targeted therapy are conducted in tertiary hospitals by performing “bedside manufacturing” (see Supplementary Material) and can easily be adopted for HIV patients. RIT can be combined with the immunoPET imaging, which would add new dimension to the developing of HIV curative drugs and to studying the mechanisms of HIV pathogenesis (47).

While we do not anticipate 2556 mAb-binding interference from the fusion inhibitors, maraviroc and enfuvirtide, which bind gp41 later in the viral life-cycle than 2556 mAb, and in a different region of gp41, they will be an important category of ART drugs to explore in the future trials. Our results using PBMCs from HIV-infected individuals suggest that complete eradication of HIV-infected cells is likely to require more than one RIT administration. A major challenge that remains is to eradicate latently infected cells. Studies in non-human primates to test RIT in combination with ART are planned.

## Author Contributions

ED, BZ, AC, and ES designed the study; DT, ED, and AMF performed the experiments; AM, FB, SZ-P, EDol, and MG provided the reagents; ED, DT, JB, and BZ analyzed the results; ED and DT wrote the manuscript; and all authors approved the final version of the manuscript.

## Conflict of Interest Statement

The authors declare that the research was conducted in the absence of any commercial or financial relationships that could be construed as a potential conflict of interest.
