# Supplementary material for: Combination of Antiretroviral Drugs and Radioimmunotherapy Specifically Kills Infected Cells from HIV-Infected Individuals
Source: Front Med (Lausanne). 2016 Sep 26;3:41. doi: 10.3389/fmed.2016.00041 (PMC5035742; doi:10.3389/fmed.2016.00041)
Supplement: Supplementary file 1 [file presentation_1.pdf]

a)

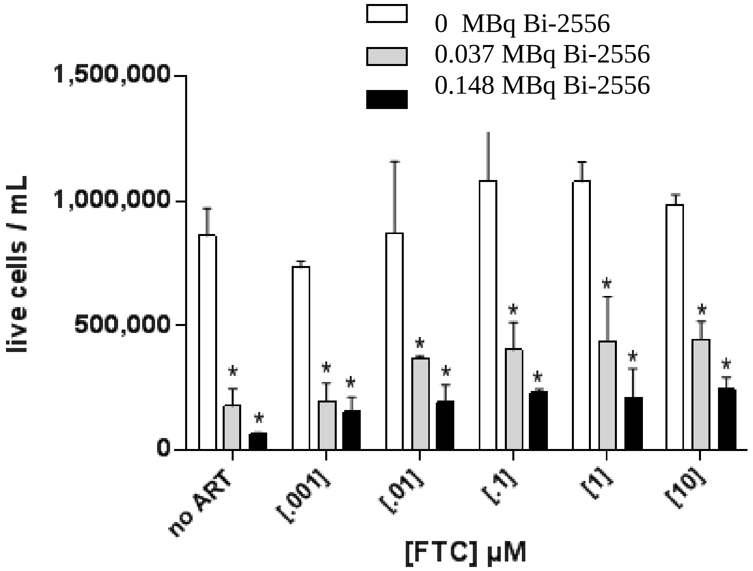
0 MBq Bi-2556
0.037 MBq Bi-2556
0.148 MBq Bi-2556
live cells / mL
1,500,000
1,000,000
500,000
0
no ART
[.001]
[.01]
[.1]
[1]
[10]
[FTC] µM

b)

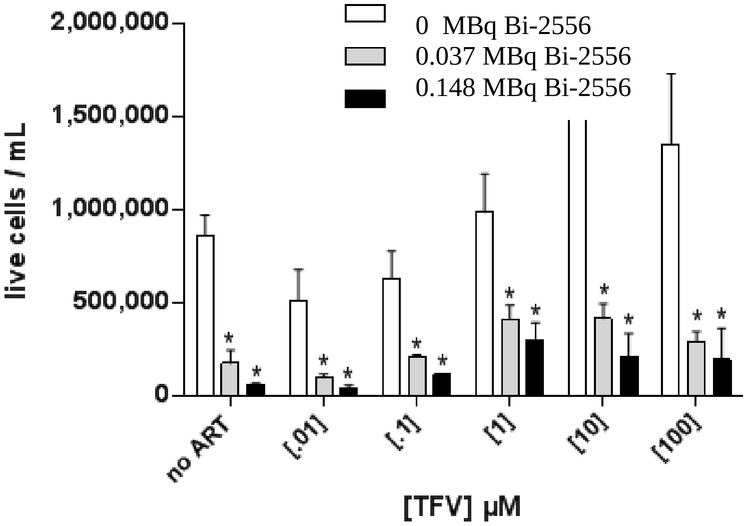
0 MBq Bi-2556
0.037 MBq Bi-2556
0.148 MBq Bi-2556
live cells / mL
2,000,000
1,500,000
1,000,000
500,000
0
no ART
[.01]
[.1]
[1]
[10]
[100]
[TFV] µM

c)

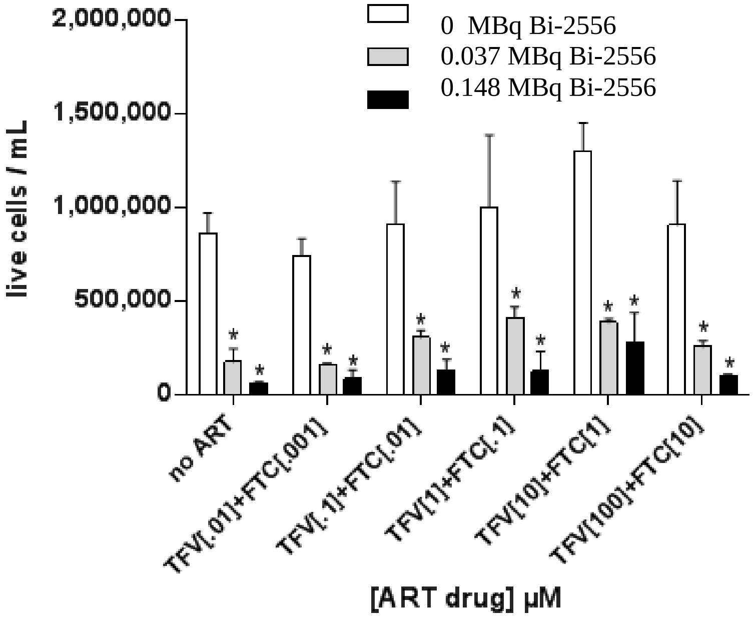
0 MBq Bi-2556
0.037 MBq Bi-2556
0.148 MBq Bi-2556
live cells / mL
2,000,000
1,500,000
1,000,000
500,000
0
no ART
TFV[.01]+FTC[.001]
TFV[.1]+FTC[.01]
TFV[1]+FTC[.1]
TFV[10]+FTC[1]
TFV[100]+FTC[10]
[ART drug] µM

Fig. 1S Killing of PBMCs infected with X4 HIV_{NL4-3} strain and treated with single NRTIs. ^{213}Bi-2556 killing of: a) emtricitabine (FTC) treated cells; b) tenofovir (TFV) treated cells; c) combined FTC and TFV treated cells. The error bars show standard deviation, experiment was performed 3 times, * denotes statistically significant p value of <0.05 compared to the 0 MBq condition within each ART group.

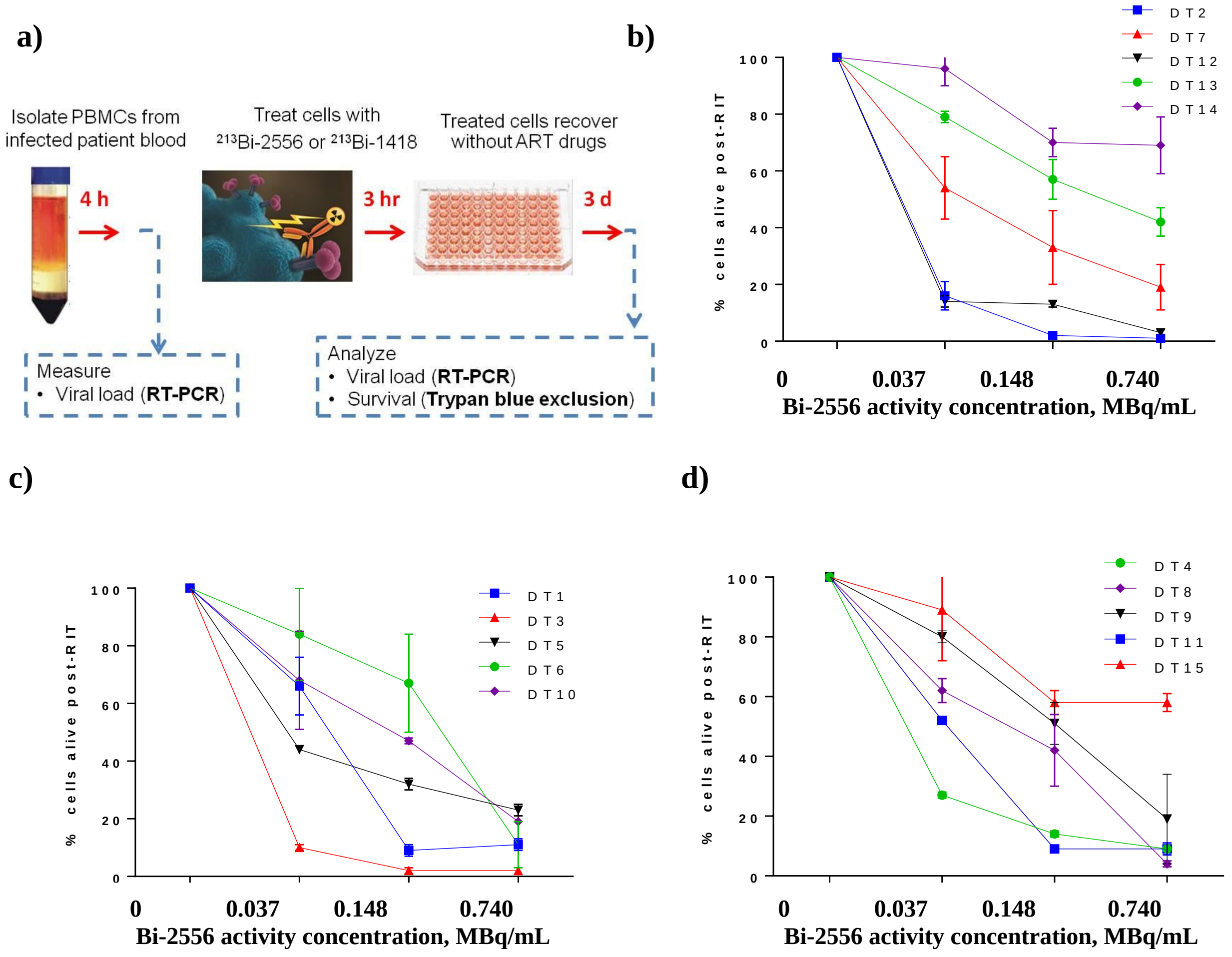

Fig. S2 ^{213}Bi-2556-induced killing of PBMCs derived from ART-treated patients: a) treatment schematic; b) TFV/FTC/EFV; c) TFV/FTC/ATZ/RTV; d) ART naïve group. The error bars show standard deviation.

Clinical preparation of ^{213}Bi-2556. For rapid preparation of radiolabeled antibodies, ^{213}Bi is eluted from a ^{225}Ac/^{213}Bi generator installed in radiopharmacy (5 min). The antibody which has already been conjugated to the CHXA" ligand and undergone all quality control procedures is radiolabeled with ^{213}Bi (5 min), purified on a disposable sterile cartridge (3 min), and the ^{213}Bi-antibody is administered to a patient in the injection room. Even if the generator cannot provide a planned single dose within one elution, the generator achieves transient equilibrium every 2 hrs which means that it can be eluted again and a patient can receive the cumulative dose within 2-3 hrs. In those departments equipped with SPECT machines capable of detecting the 440 keV high energy photons emitted by ^{213}Bi, patients are also imaged during the radiolabeled antibody administration to monitor uptake at the tumor/infection site. The imaging of ^{213}Bi-labeled mAb while it was being administered to patients was performed by Sgouros et al (Suppl. Ref. 1), and they were recording uptake in spleen (the patients were leukemic) and liver as early as 15 min after the injection. However, in a trial where ^{213}Bi-labeled mAb was given to melanoma patients, the quality of images was not sufficient for dosimetry calculations (Suppl. Ref. 2). As HIV resembles in a way single cell cancers such as leukemia, we hope to see the fast uptake of the ^{213}Bi-2556 in the lymph nodes, GALT tissue etc. which might contain infected cells.

Supplemental References:

1)Sgouros G. et al. Pharmacokinetics and Dosimetry of an α-Particle Emitter Labeled Antibody: 213Bi-HuM195 (Anti-CD33) in Patients with Leukemia. J Nucl. Med 1999; 40:1935-1946

2)Raja C. et al. Interim Analysis of Toxicity and Response in Phase 1 Trial of Systemic Targeted Alpha Therapy for Metastatic Melanoma Cancer Biology & Therapy 2007; 6:6, 846-852.
